# Effects of ChatGPT’s AI capabilities and human-like traits on spreading information in work environments

**DOI:** 10.1038/s41598-024-57977-0

**Published:** 2024-04-02

**Authors:** Hyeon Jo, Do-Hyung Park

**Affiliations:** 1 Headquarters, HJ Institute of Technology and Management, 71 Jungdong-ro 39, Bucheon-si, Gyeonggi-do 14721 Republic of Korea; 2 Graduate School of Business IT, Kookmin University, 77, Jeongneung-ro, Seongbuk-gu, Seoul, 02707 Republic of Korea

**Keywords:** ChatGPT, Knowledge Acquisition, Knowledge Application, Word-of-Mouth, Office Workers, Environmental social sciences, Mathematics and computing

## Abstract

The rapid proliferation and integration of AI chatbots in office environments, specifically the advanced AI model ChatGPT, prompts an examination of how its features and updates impact knowledge processes, satisfaction, and word-of-mouth (WOM) among office workers. This study investigates the determinants of WOM among office workers who are users of ChatGPT. We adopted a quantitative approach, utilizing a stratified random sampling technique to collect data from a diverse group of office workers experienced in using ChatGPT. The hypotheses were rigorously tested through Structural Equation Modeling (SEM) using the SmartPLS 4. The results revealed that system updates, memorability, and non-language barrier attributes of ChatGPT significantly enhanced knowledge acquisition and application. Additionally, the human-like personality traits of ChatGPT significantly increased both utilitarian value and satisfaction. Furthermore, the study showed that knowledge acquisition and application led to a significant increase in utilitarian value and satisfaction, which subsequently increased WOM. Age had a positive influence on WOM, while gender had no significant impact. The findings provide theoretical contributions by expanding our understanding of AI chatbots' role in knowledge processes, satisfaction, and WOM, particularly among office workers.

## Introduction

ChatGPT, developed by OpenAI, has emerged as a powerful conversational artificial intelligence (AI) system that leverages the capabilities of machine learning and natural language processing^1,2^. This system has gained significant traction in the workspace, providing office workers with an efficient and effective means of completing a variety of tasks, from drafting emails to assisting with research^3,4^. ChatGPT's ability to produce human-like text enables it to facilitate multiple functions in the workplace^5^. For instance, it assists in drafting content, provides answers to queries, and aids in brainstorming sessions^6^. This wide range of applications underscores its increasing integration into daily work tasks, which necessitates a deeper understanding of its interaction dynamics with users. ChatGPT and similar AI-driven conversational agents are increasingly being used in workplace settings, transforming how office workers perform their tasks and communicate. According to the latest data as of March 2023, approximately 8.2% of employees across global organizations have experimented with utilizing ChatGPT within their work environment at least once^7^.

Interestingly, the widespread awareness and adoption of ChatGPT in workplace environments can largely be attributed to informal channels of communication rather than traditional advertising methods. Despite OpenAI not engaging in extensive Above-The-Line (ATL) or Below-The-Line (BTL) advertising campaigns for ChatGPT, knowledge of its existence and usefulness has proliferated, primarily through word-of-mouth among individuals. This phenomenon is particularly pronounced among younger generations, who are not only more open to embracing innovative services but also actively use social media as their primary medium for information exchange and communication. This trend highlights the critical role of word-of-mouth in the acceptance and diffusion of new services like ChatGPT, a factor that has often been overlooked in traditional marketing approaches but is evidently powerful in the digital era.

Word-of-mouth (WOM) plays a crucial role in shaping users' perceptions and decisions to adopt new technologies^8^. In the context of ChatGPT, it becomes a potent tool for capturing the overall user experience. It allows us to assess not just user satisfaction but also their likelihood of recommending the technology to others, which is critical for its adoption and diffusion in office settings^9^. Examining WOM among office workers using ChatGPT is particularly relevant in the current times when the workplace landscape is increasingly digital and remote due to global conditions. As reliance on AI-driven tools intensifies, understanding how users narrate their experiences can provide insights into the technology's strengths and areas of improvement.

Recent advancements in generative AI have become a focal point of considerable research. Central to these developments is ChatGPT, which has significantly contributed to the commercialization of generative AI, spurring a wave of active research in this field. Research on generative AI is currently delving into three main areas, each contributing to the advancement of this evolving field. The first area focuses on algorithmic improvements within computer science, aiming to refine the underlying models and techniques used in generative AI^10^. This includes developments in model architectures like GANs and VAEs, as well as transformer-based models such as GPT. The second research area is centered around the exploration of prompts for problem-solving using generative AI within computer language and decision science^11^. Researchers are actively investigating how prompts can be strategically employed to influence and guide generative AI systems, aiming to optimize their performance. This is particularly relevant in decision science, where the focus is on leveraging generative AI to assist in complex decision-making processes. The third area of research delves into understanding user acceptance and satisfaction with generative AI from the perspective of user behavior^12,13^. This involves studying factors that impact user trust, satisfaction, and overall usability of generative AI applications. Researchers are keen on deciphering user interactions, feedback, and preferences to inform the design and implementation of generative AI systems in practical, real-world scenarios. Within the scope of these research areas, this study specifically focuses on the third aspect, examining the factors influencing user satisfaction with generative AI, thereby contributing to a deeper understanding of how users interact with and perceive these advanced technologies.

Previous research in the context of AI and workplace interactions has often overlooked the complexity of the relationship between AI features, perceived value, satisfaction, and WOM behaviors. The specific roles of various AI intelligence elements such as system update, memorability, and non-language barriers and their interplay with user knowledge factors (knowledge acquisition and application) need a closer look. Furthermore, the comprehensive examination of the influence of human-like personality on AI utilitarian value, satisfaction, and WOM has not been thoroughly explored in a workplace context.

This study offers a detailed investigation into the correlation between the characteristics of AI systems and the behaviors exhibited by users, highlighting the interconnected roles of system intelligence, user knowledge, and human-like personality in driving user satisfaction and WOM in the workplace. As well, the study contextualizes these relationships specifically within a workplace setting, an area that has not received adequate attention in ChatGPT research. Further, it applies an integrative approach, combining various elements (intelligence, knowledge, and human factors) to develop a holistic model of user interaction with AI, thus filling gaps in the existing body of literature. In addition, the study is one of the first to examine these relationships in the context of ChatGPT, an AI tool with widespread use in various sectors, thereby contributing valuable insights into the functioning and acceptance of AI technology in the workplace. Based on the aforementioned research background, the objectives of this study are:

1. To develop an integrative model encompassing intelligence, knowledge, human, and innovation factors, to better explain user behaviors in the workplace, specifically relating to AI technology usage.
2. To investigate how intelligence elements of AI (system update, memorability, non-language barriers) affect knowledge acquisition and application of the user, in a workplace setting.
3. To understand the role of the human-like personality of ChatGPT on perceived utilitarian value and satisfaction
4. To confirm the roles of utilitarian value and satisfaction as antecedents of WOM, thus explaining user behaviors in the workplace.

By achieving the four research objectives outlined previously, this study can address the research gap from the following three perspectives. Firstly, while there are emerging studies on the acceptance of ChatGPT, there is a noticeable dearth of research exploring the complete path from usage to satisfaction. Previous studies have tended to be fragmented, focusing on isolated aspects of AI chatbot interaction. Our study aims to bridge this gap by providing a comprehensive analysis of how the features of ChatGPT influence knowledge processes and subsequently lead to user satisfaction and word-of-mouth (WOM) in office settings. This holistic approach is seldom seen in current literature. Secondly, we also recognize the lack of research on the detailed multi-stage mechanism by which features of ChatGPT, such as system updates and human-like personality traits, contribute to knowledge acquisition and creation, thereby delivering true value and influencing user satisfaction. Our study fills this gap by systematically examining these relationships, providing a deeper understanding of the role of ChatGPT in the knowledge process within office environments. Lastly, the interconnection between value-based satisfaction and WOM activities has not been sufficiently explored in the context of AI chatbots like ChatGPT. Our study uniquely contributes to this area by demonstrating how the utilitarian value derived from knowledge acquisition and application can lead to increased satisfaction and subsequently trigger WOM among office workers. This aspect of our research underscores the practical implications of AI integration in digital and remote work settings.

The structure of this paper unfolds in six main sections. After the introductory section, "Theoretical background and hypothesis development" dives into the theoretical background and hypothesis development, drawing on relevant literature surrounding AI, user interaction, utilitarian value, satisfaction, and WOM. Following that, "Research methodology" describes the methodology employed in this study, detailing the research design, sampling, data collection, and analytical procedures. The subsequent section, "Analysis and results", provides an analysis of the results, interpreting and quantifying the data gathered. "Discussion" then engages in a comprehensive discussion of these results, providing insights, explaining anomalies, and connecting findings with pre-existing knowledge in the field. The paper concludes with "Conclusion", encapsulating the study's key findings, potential implications, and suggestions for future research in this arena.

### Theoretical background and hypothesis development

The research model of this study is designed to investigate the various elements influencing WOM behavior among office workers using the ChatGPT AI system in their workplace. The model focuses on three primary intelligence factors: system update, memorability, and non-language barriers. AI encompasses a wide range of intelligent capabilities (intelligence factors), including system updates, memory management, and language proficiency. System update helps in understanding the perceived value and acceptance of the updated system features by the users. Users' perception of system updates may influence their overall satisfaction, their attitudes toward the technology, and ultimately, their intention to continue using it^14^. The memorability of an AI-driven technology like ChatGPT carries significant importance for user engagement, retention, and overall experience^15,16^. It is a key facet of user experience and has been shown to affect technology adoption and usage behavior^17^. In the realm of conversational AI like ChatGPT, memorability can be associated with how impactful or meaningful a user finds the generated responses. This might include the system's ability to deliver accurate, novel, or emotionally resonant replies. High memorability can enhance user satisfaction, encourage repeat use, and foster a sense of connection or rapport with the system^18^. Non-language barriers hold significant importance for understanding and enhancing the user experience^14^. In the current globalized environment, the workspace is becoming increasingly multilingual. Organizations are embracing diversity, making multilingual communication a common scenario in office environments (Lauring, 2008). If an AI system like ChatGPT can understand languages from different countries, seamlessly switch between languages, and cater to multilingual communication needs, it can be an invaluable asset for companies seeking to enhance customer loyalty^19^. System update, memorability, and non-language barriers are representative of the AI's intelligent elements, having a considerable impact on user interaction in the workplace. These factors (e.g. system update, memorability, and non-language barriers) are integral in facilitating knowledge acquisition and knowledge application.

Knowledge factors, specifically knowledge acquisition and knowledge application, are crucial elements when considering user behaviors with AI technology such as ChatGPT^20^. These elements are derived from the cognitive perspective of human–computer interaction (HCI) studies, which emphasize the mental processes such as perception, memory, learning, and problem-solving in using technologies^21^. Essentially, they encapsulate how users understand and utilize the information provided by the AI system in their tasks. Users' ability to acquire knowledge from ChatGPT is particularly relevant in the workplace, as users must understand the system's outputs to leverage the AI's capabilities in their tasks. For instance, ChatGPT can provide information and insights to users in various domains such as project management^22^, drafting emails^23^, or research tasks^24^, thereby aiding the user in acquiring knowledge. Knowledge application, on the other hand, refers to the utilization of the acquired knowledge in relevant situations or tasks^25^. The ability to apply the acquired knowledge is also crucial in the context of using AI in the workplace, as it signifies the system's utility in assisting users in their job-related tasks^26^. The model posits that the knowledge factors drive the perceived utilitarian value and satisfaction derived from the AI system.

Additionally, the employment of a human-like personality in AI systems, particularly in the workplace context, is a crucial element to consider. This is based on the Person-Artifact-Task (PAT) model, which suggests that the interplay between the person (user), the artifact (AI), and the task influences the acceptance and use of technology^27^. In line with this, AI systems like ChatGPT with a more human-like personality can foster an interactive environment, enhancing user satisfaction and engagement^28^. Workplace interactions require rational and logical communication, and AI with a human-like personality could exhibit these qualities effectively. ChatGPT, for instance, can understand, generate, and converse in natural language, thereby mimicking human-like conversation rationally and logically. Furthermore, the concept of human-like personality in AI systems has been extensively validated to affect perceived value and satisfaction, the key antecedents of WOM^14,29,30^. Users who perceive an AI system to exhibit human-like personality traits can develop a sense of rapport, trust, and ultimately satisfaction with the system^30–32^. The human-like personality of the ChatGPT AI system is incorporated into the model as a factor that further influence perceived value and satisfaction.

In the context of ChatGPT usage within the workplace, the roles of utilitarian value and satisfaction become highly relevant due to their significant impacts on user behaviors, particularly WOM. When workers perceive a high utilitarian value in a tool like ChatGPT—through its abilities to increase productivity, facilitate tasks, or provide precise information—they are likely to communicate these positive experiences and advocate the technology to their peers^33,34^. Similarly, when users experience satisfaction with their interactions with a system such as ChatGPT, they are more likely to engage in positive WOM by recommending it to others in their network^35^. In the context of workplace technology, satisfaction often translates into how well the system assists users in their tasks, how easy it is to use, and how reliably it functions—factors that significantly shape the users' WOM behaviors. The model proposes that the perceived utilitarian value and satisfaction derived from the AI system influence WOM behavior directly. It directly affects task performance and can lead to satisfaction and further WOM recommendation of the system.

**Figure 1:**
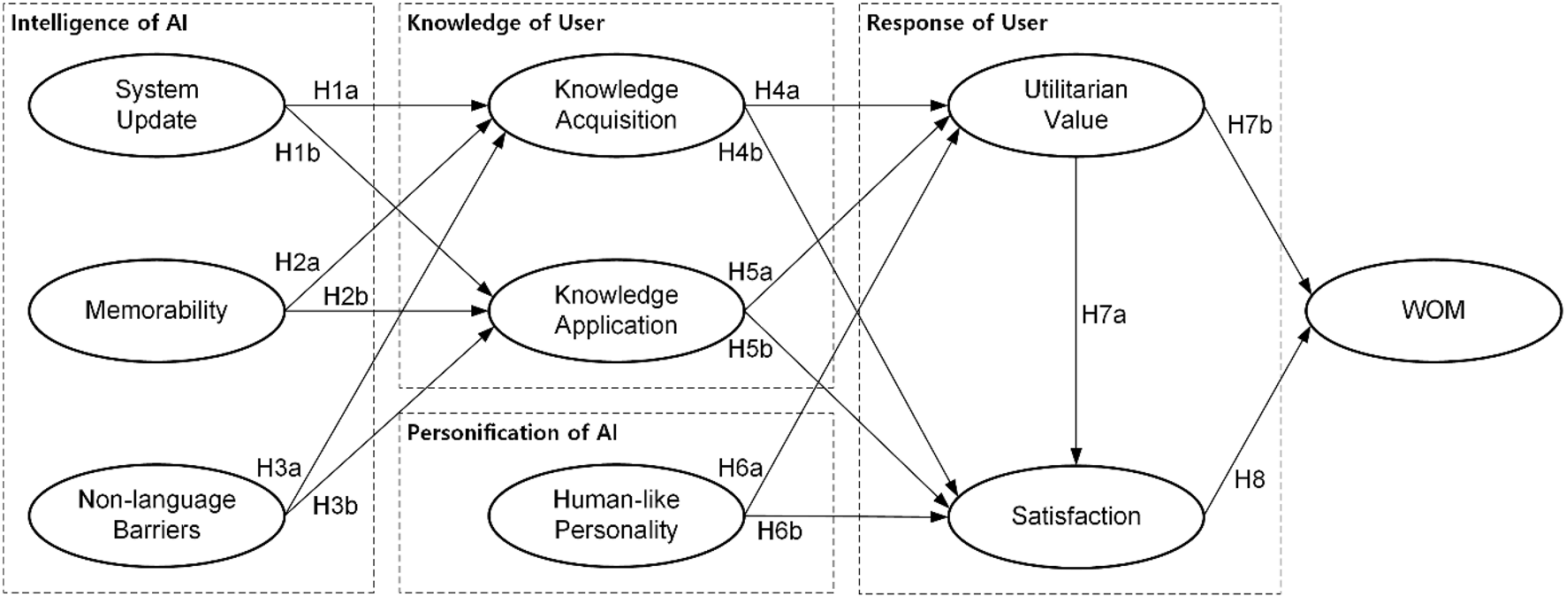
Conceptual Model.

Consequently, the unique characteristics of ChatGPT significantly influence the knowledge factors, which are critical in explaining user behaviors towards AI systems in the workplace. These cognitive processes, shaped by the distinct attributes of ChatGPT, determine how users perceive and utilize the technology, ultimately affecting their satisfaction, task performance, and WOM behaviors. The research model thus offers a comprehensive view of the factors driving WOM behavior related to the use of the ChatGPT AI system in the workplace, drawing on various theoretical foundations to explain the complex interplay of these elements. Figure 1 shows the conceptual model.

### Intelligence of AI

#### System update

System updates often encompass software modifications to improve performance, fix bugs, add new features, or enhance user experience. Upgrades can significantly influence the functionality, usability, and overall effectiveness of AI-based systems like ChatGPT^36^. A number of studies have posited a positive relationship between the advancement of AI systems and the knowledge management processes of users^37–40^. Knowledge acquisition and knowledge applications are integral components of knowledge management, which significantly impacts organizational performance^41^. System upgrades that involve improved natural language processing and understanding can significantly enhance AI systems' ability to understand and process complex information, which can then be effectively communicated to users^42^. Such updates can also improve the system's ability to interact and engage with users in a more human-like manner, which can motivate users to explore and utilize the system's knowledge-related functionalities^43^. Enhanced AI functionalities, improved information processing, and a more interactive user experience can support the workers in their continuous learning endeavors and facilitate the application of this knowledge to their daily tasks. Thus, this study suggests the following hypotheses:

##### H1a

System update of ChatGPT has a positive impact on knowledge acquisition of office workers.

##### H1b

System update of ChatGPT has a positive impact on knowledge application of office workers.

#### Memorability

Memorability refers to ChatGPT's capability to recall user needs, preferences, and past interactions^14^. This aspect of the AI system's design and functionality can greatly influence user engagement, satisfaction, and overall experience. Several studies in the field of human–computer interaction and AI indicate that system memorability can significantly facilitate these knowledge management processes^44,45^. When a system remembers and recognizes user needs and preferences, it can provide more personalized, relevant, and thus, beneficial information. This, in turn, enhances knowledge acquisition as users are more likely to engage with and absorb information that directly caters to their needs. In addition, memorability can aid in knowledge application. When a system recalls past interactions and user preferences, it can offer insights or suggestions relevant to the user's tasks^46^. This allows for the effective application of the knowledge provided by the system, as it aligns with the user's context, needs, and preferences. Given that office workers often deal with tasks requiring a wide range of knowledge and rapid problem-solving, a system with high memorability can be particularly useful. It can aid in curating and providing personalized information, thus enhancing the processes of knowledge acquisition and application. Therefore, this study proposes the following hypotheses:

##### H2a

Memorability of ChatGPT has a positive impact on knowledge acquisition of office workers.

##### H2b

Memorability of ChatGPT has a positive impact on knowledge application of office workers.

#### Non-language barriers

Non-language barriers pertain to the AI system's ability to comprehend, translate, and communicate in multiple languages^14^. A system's multilingual capability can serve to break down language barriers and promote more seamless communication between users and the AI^47^. Research has indicated that language plays a crucial role in knowledge acquisition and application processes^48^. An AI system that transcends language barriers, like ChatGPT, can greatly facilitate these processes. With the ability to understand and communicate in multiple languages, it can provide more accessible and comprehensible information to users^14^. This potentially enhances knowledge acquisition by ensuring that users can fully understand the information provided. Moreover, a multilingual AI system can offer personalized knowledge application solutions tailored to the user's language and cultural context^49^. This can significantly facilitate the application of knowledge in a user's daily tasks and problem-solving activities. Thus, this study suggests the following hypotheses:

##### H3a

Non-language barriers of ChatGPT have a positive impact on knowledge acquisition of office workers.

##### H3b

Non-language barriers of ChatGPT have a positive impact on knowledge application of office workers.

### Knowledge of user

#### Knowledge acquisition

Knowledge acquisition involves the acquisition of new knowledge and the subsequent expansion of that knowledge when new information is obtained^50^. This plays a crucial role in shaping a user's interaction with and perception of AI systems^20^. Prior research indicates that effective knowledge acquisition can significantly contribute to perceived usefulness^50,51^. By providing relevant, tailored information, AI systems like ChatGPT can enhance users' task performance and efficiency, thereby increasing the utilitarian value derived from the system. Knowledge acquisition can lead to higher levels of user satisfaction^52^. When users can easily and effectively acquire the information they need, they are likely to feel more satisfied with the system providing that information. Both utilitarian value and satisfaction are crucial outcomes for users interacting with AI systems like ChatGPT. In an office setting, acquiring the necessary knowledge effectively can help users complete their tasks more efficiently, increasing the utilitarian value of the system. Concurrently, it can enhance user satisfaction by meeting or exceeding user expectations. Therefore, this study suggests the following hypotheses:

##### H4a

Knowledge acquisition from ChatGPT positively impacts the utilitarian value derived from it.

##### H4b

Knowledge acquisition from ChatGPT positively impacts satisfaction with it.

#### Knowledge application

Knowledge application is defined as the process of using acquired knowledge in decision-making, problem-solving, or task execution^25^. There's a body of research suggesting that the effective application of knowledge can substantially enhance the perceived utilitarian value of a system^26,53^. This is because the ability to apply knowledge obtained from a system, such as ChatGPT, can improve task performance and efficiency. Effective knowledge applications can lead to higher user satisfaction, as reported in several studies^54–56^. The ability to apply acquired knowledge can enhance the user experience, meeting or exceeding user expectations and leading to higher levels of satisfaction. Both utilitarian value and satisfaction are essential outcomes for users interacting with AI systems. Knowledge applications can help users leverage the information provided by the system in a practical and meaningful way, increasing utilitarian value. At the same time, it can enhance user satisfaction by making user interaction more effective and rewarding. Consequently, this study suggests the following hypotheses:

##### H5a

Knowledge application via ChatGPT has a positive impact on utilitarian value derived from it.

##### H5b

Knowledge application via ChatGPT has a positive impact on satisfaction with it.

### Personification of AI

#### Human-like personality

The human-like personality of an AI system refers to its ability to exhibit traits similar to human characteristics, such as having a unique identity or characteristics^57^. Studies have shown that users tend to derive greater utilitarian value from systems that exhibit human-like personality traits, as they are perceived to be more relatable and interactive, thereby enhancing the user's efficiency and task performance^33,58^. Prior studies have suggested that AI systems with human-like personalities can significantly improve user satisfaction^28^. Users tend to feel more satisfied when interacting with a system that shows human-like traits as it fosters a more personalized and engaging experience^14,59^. Both utilitarian value and satisfaction are important outcomes of user interactions with AI systems like ChatGPT. The presence of a human-like personality can enhance the user's ability to derive practical benefits from the system and increase satisfaction by making interactions more enjoyable and engaging. Thus, this study suggests the following hypotheses:

##### H6a

Human-like personality in ChatGPT has a positive impact on utilitarian value derived from it.

##### H6b

Human-like personality in ChatGPT has a positive impact on satisfaction with it.

### Response of user

#### Utilitarian value

Utilitarian value is understood as the functional or practical benefits a user obtains from a product or service^60^. It often pertains to increased productivity, efficiency, or improved task performance^61^. Several studies have validated the positive association between utilitarian value and satisfaction, arguing that when users derive practical benefits from a system, it significantly enhances their satisfaction levels^62–64^. The utilitarian value obtained from a service or product often prompts users to share their positive experiences with others. This relationship has been established in previous research, suggesting that a high utilitarian value often results in positive WOM^33,34^. Utilitarian value can increase satisfaction by providing practical benefits and encouraging positive WOM, which can in turn enhance the overall user experience and system adoption. Thus, this study suggests the following hypotheses:

##### H7a

Utilitarian value derived from ChatGPT has a positive impact on satisfaction with it.

##### H7b

Utilitarian value derived from ChatGPT has a positive impact on WOM.

#### Satisfaction

Satisfaction refers to the degree to which a user's expectations about a product or service are met or exceeded^65^. It is an essential component in determining users' overall perceptions of the quality of a product or service^66^. The relationship between satisfaction and WOM has been extensively studied and well-established^67,68^. Satisfied customers are more likely to share their positive experiences with others, influencing their decision-making process^32,69^. The relationship between satisfaction and WOM is particularly significant in the context of AI systems like ChatGPT. If users are satisfied with their experiences, they are more likely to recommend the service to others, contributing to its wider acceptance and adoption. Thus, this study suggests the following hypothesis:

##### H8

Satisfaction has a positive impact on WOM.

### Control variables

Age and gender are commonly considered control variables in research, as they may impact the relationship between independent and dependent variables^70–72^. This is because age and gender often influence individuals' behaviors, perceptions, and attitudes, including their reactions to technologies and AI systems like ChatGPT. Considering age and gender as control variables can help isolate the effects of these demographic factors, providing a clearer understanding of the relationship between the primary variables of interest.

## Research Methodology

All methods were performed in accordance with the Declaration of Helsinki.

### Measurement instrument

The process of instrument development began with an extensive review of the literature to identify suitable items for each construct. The items for the constructs of system update, memorability, non-language barriers, and human-like personality were adapted from Chen et al.^14^, who conducted an extensive study on the quality measurements of AI chatbots. The items for knowledge acquisition and knowledge application were adapted from the study by Al-Sharafi et al.^20^. The items for utilitarian value were adapted from Kim and Oh^73^ and the items for satisfaction from Nguyen et al.^74^. The WOM construct items were adapted from Mishra and Shukla^75^. Finally, each construct's items were measured using a seven-point Likert scale ranging from 1 (strongly disagree) to 7 (strongly agree), except for the control variables. Gender was categorized as 1 for males and 2 for females, and age was recorded as collected. Table 1 details the list of constructs and items.

**Table 1:** List of Constructs and Items.

Construct	Items	Mean	Reference
System update	SUP1	I can sense that ChatGPT is constantly upgrading	Chen et al.^14^
	SUP2	I feel that ChatGPT is becoming more advanced	
	SUP3	The functionality of ChatGPT has been enhanced	
Memorability	MMR1	The service system efficiently remembers my needs and preferences	Chen et al.^14^
	MMR2	I don't have to relay my requirements to the service system repeatedly	
	MMR3	Even when I haven't used ChatGPT for a while, it still remembers my preferences and needs upon my return	
Non-language barriers	NLB1	ChatGPT understands languages from different countries	Chen et al.^14^
	NLB2	ChatGPT can switch languages seamlessly	
	NLB3	ChatGPT caters to my multilingual communication needs	
Knowledge acquisition	KAQ1	ChatGPT allows me to generate new knowledge based on my existing knowledge	Al-Sharafi et al.^20^
	KAQ2	ChatGPT enables me to access knowledge through various resources	
	KAQ3	ChatGPT assists me in acquiring knowledge that suits my needs	
Knowledge application	KAP1	ChatGPT provides instant access to a variety of knowledge types	Al-Sharafi et al.^20^
	KAP2	ChatGPT enables me to integrate different types of knowledge	
	KAP3	ChatGPT can aid in managing course materials more effectively within the university	
Human-like personality	HUP1	ChatGPT exhibits personality traits similar to those of humans	Chen et al.^14^
	HUP2	I sense that ChatGPT has its unique personality	
	HUP3	The personality of ChatGPT is comparable to a human's	
Utilitarian value	UTV1	Compared to the fee I pay (KRW 0 for the free version), the use of ChatGPT provides excellent value for money	Kim and Oh^73^
	UTV2	Considering the effort I invest, the use of ChatGPT is beneficial to me	
	UTV3	Relative to the time I spend, the use of ChatGPT is worthwhile	
Satisfaction	SAT1	ChatGPT has met my expectations	Nguyen et al.^74^
	SAT2	ChatGPT efficiently fulfills my needs, such as seeking information or completing transactions	
	SAT3	I am satisfied with the support provided by ChatGPT	
WOM	WOM1	I'll spread positive word of mouth about ChatGPT	Mishra and Shukla^75^
	WOM2	I'll recommend ChatGPT to my friends	
	WOM3	I'll encourage my friends to use or purchase ChatGPT	
Control variables	Gender is categorized as 1 for males and 2 for females Age is recorded as collected		

The questionnaire was structured in two sections. The first section of the questionnaire consisted of questions aimed at identifying the respondent's demographic information, including gender, age, and position. This approach was employed to ensure that the sample well-represented the broader population. The second part of the questionnaire consisted of multiple items that were designed to measure each construct. These constructs included system update, memorability, non-language barriers, knowledge acquisition, knowledge application, human-like personality, utilitarian value, satisfaction, and WOM. Special attention was given to ensuring that the items were clear, concise, and unambiguous. The language and terminology used in the questionnaire were carefully chosen to be easily understood by the respondents without any specialized knowledge. A balance was also struck in the questionnaire length to avoid respondent fatigue while ensuring comprehensive coverage of all the constructs.

Before the actual data collection, the questionnaire underwent an expert review process and a pilot test to assess its validity and reliability. An essential step in ensuring the validity of the research questionnaire was the conduct of an expert review. A panel of three experts in the field of information systems, office management, and survey design was selected to evaluate the instrument's validity. These experts were chosen based on their extensive knowledge and experience in the relevant fields and their understanding of questionnaire design and methodology. The experts were asked to assess the content and construct validity of the questionnaire, which included examining the clarity, relevance, and comprehensiveness of the items concerning the constructs they were meant to measure. Each expert was also asked to provide suggestions for improving the questionnaire, such as rewording unclear items or adding new ones that could better capture the constructs of interest. The feedback from the expert review was invaluable in refining the questionnaire. All the suggested changes were carefully evaluated and incorporated into the final instrument wherever appropriate. For instance, based on the experts' input, some items were rephrased for better clarity, while others were excluded to prevent redundancy. Moreover, a few new items were added to ensure the comprehensive coverage of the constructs. Subsequently, a pilot test was carried out with a sample of 20 office workers, which further ensured the clarity and comprehension of the items.

This robust instrument development process ensured that the measures used in the study were both valid and reliable, adequately capturing the constructs of interest.

### Subject and data collection

The data for this study was collected via a survey which was conducted by a professional polling company, Hankook Research. Given the objective of the research to understand workers' usage and intention to use ChatGPT, the survey targeted workers across various industries. The sampling method used in this study is stratified sampling, which is a type of probability sampling method. Stratified sampling is widely recognized for its ability to improve the representativeness and generalizability of findings, especially when there is heterogeneity within the population^76^. The population in this study was divided into strata based on two variables: gender and job position. Age groups were also considered in forming the strata, with a focus on the 20s to 40s age group as they are the primary users of ChatGPT in the workplace. The process of distributing and collecting the survey took place over approximately ten days, starting from the end of May and extending into early June of 2023.

The sample comprised 347 respondents, carefully selected to ensure adequate representation across demographics. As illustrated in Table 2, the gender distribution was balanced with males accounting for 50.4% (175 respondents) and females making up 49.6% (172 respondents) of the total respondents. In terms of age, respondents were evenly distributed across the age groups of 20, 30, and 40s, each group representing approximately a third of the total sample. This age range was selected as they are considered to be the primary users of AI tools like ChatGPT in the workplace. The sample also ensured representation across different job positions. The largest group of respondents were Assistant Managers (27.4%, 95 respondents), followed by Clerks (25.1%, 87 respondents), and Managers (20.7%, 72 respondents). Senior Managers accounted for 13.5% (47 respondents), Executive Managers made up 11.0% (38 respondents), and Directors and Representatives each represented 1.2% (4 respondents each) of the total sample. The distribution across different demographics not only provides a comprehensive view of the use of ChatGPT among workers but also allows for comparison across different groups, thus enhancing the generalizability of the study's findings.

**Table 2:** Demographic features of respondents.

Demographics	Item	Subjects (N = 347)	
		Frequency	Percentage
Gender	Male	175	50.4%
	Female	172	49.6%
Age	20s	116	33.4%
	30s	116	33.4%
	40s	115	33.1%
Position	Clerk	87	25.1%
	Assistant manager	95	27.4%
	Manager	72	20.7%
	Senior manager	47	13.5%
	Executive manager	38	11.0%
	Director	4	1.2%
	Representative	4	1.2%

### Ethical approval

This study was approved by an institutional review board of HJ Institute of Technology and Management.

### Informed consent

Informed consent and Consent to participate was obtained from all individual participants included in the study.

### Analysis and results

The structural equation modeling (SEM) technique, specifically Partial Least Squares (PLS), was employed to analyze the data. PLS-SEM was chosen due to its ability to handle complex models with many constructs and indicators, its robustness to violations of normality, and its applicability to both theory testing and development^77^. Moreover, PLS-SEM is suitable for exploratory research, such as this study, that aims to explain the key drivers of a dependent variable^78^. This section presents the validation results for common method bias, the measurement model, and the structural model.

### Common method bias (CMB)

To ensure the validity of our findings and mitigate the potential threat of common method bias, we conducted Harman's single-factor test. The analysis yielded a single construct value of 48.891%, indicating that common method bias is not a substantial issue in our study as the value does not surpass the 50% threshold^79^. Further, we assessed the variance inflation factor (VIF) to measure the severity of multicollinearity in the regression analysis. The VIF values ranged from 1.020 to 2.503. As none of the VIF values exceeded the threshold of 3.3^80^, it suggests that multicollinearity is not an issue in our study. These results suggest that the common method bias and multicollinearity are not significant concerns in our study, and thus, the findings are robust and reliable.

### Measurement model

This research used a two-step approach recommended by Anderson and Gerbing^81^ to test the reliability, convergent validity, and discriminant validity of the measurement model. For reliability analysis, Cronbach's Alpha and Composite Reliability (CR) were used. As shown in Table 3, all constructs exhibited acceptable reliability with Cronbach's Alpha values ranging from 0.827 to 0.943, which exceeded the recommended threshold of 0.7^82^ Likewise, all constructs also had CR values above the acceptable level of 0.7^82^, ranging from 0.897 to 0.964. These results confirmed the reliability of our measurement scales.

**Table 3:** Test results of reliability and validity.

Sort	Items	Measures	St. Dev	Factor loading	Cronbach's alpha	CR	AVE
System update	SUP1	4.703	1.273	0.913	0.897	0.936	0.829
	SUP2	4.850	1.215	0.917			
	SUP3	4.844	1.209	0.901			
Memorability	MMR1	4.833	1.162	0.894	0.856	0.911	0.774
	MMR2	4.481	1.331	0.863			
	MMR3	4.481	1.276	0.883			
Non-language barriers	NLB1	5.069	1.142	0.881	0.868	0.919	0.791
	NLB2	5.049	1.090	0.903			
	NLB3	4.916	1.140	0.884			
Knowledge acquisition	KAQ1	4.994	1.279	0.879	0.889	0.931	0.818
	KAQ2	5.104	1.159	0.931			
	KAQ3	5.032	1.135	0.904			
Knowledge application	KAP1	5.239	1.133	0.876	0.827	0.897	0.744
	KAP2	5.101	1.161	0.889			
	KAP3	4.859	1.129	0.821	0.897	0.936	0.830
Human-like personality	HUP1	4.380	1.366	0.896			
	HUP2	4.354	1.401	0.924			
	HUP3	4.285	1.443	0.912	0.909	0.943	0.847
Utilitarian value	UTV1	5.303	1.160	0.880			
	UTV2	5.161	1.107	0.947			
	UTV3	5.130	1.180	0.931	0.907	0.942	0.843
Satisfaction	SAT1	4.709	1.146	0.909			
	SAT2	4.890	1.159	0.917			
	SAT3	4.934	1.178	0.929	0.943	0.964	0.898
WOM	WOM1	4.977	1.272	0.949			
	WOM2	4.971	1.263	0.956			
	WOM3	4.997	1.278	0.938			

Convergent validity was confirmed through factor loadings and the Average Variance Extracted (AVE). The factor loadings for all items were significant and exceeded the recommended level of 0.7^82^, with values ranging from 0.821 to 0.956. Moreover, the AVE of all constructs ranged from 0.744 to 0.898, which surpassed the 0.5 thresholds^83^, further demonstrating convergent validity.

Discriminant validity was verified through Fornell-Larcker Criterion and Heterotrait-Monotrait (HTMT) Ratio. As shown in Table 4, the square roots of the AVEs (diagonal elements) were greater than their corresponding off-diagonal elements, thereby confirming discriminant validity per the Fornell-Larcker Criterion^83^.

**Table 4:** Construct Correlations and Discriminant Validity.

Constructs	1	2	3	4	5	6	7	8	9
1. System update	0.910								
2. Memorability	0.589	0.880							
3. Non-language barriers	0.450	0.531	0.889						
4. Knowledge acquisition	0.602	0.527	0.466	0.905					
5. Knowledge application	0.660	0.607	0.520	0.726	0.862				
6. Human-like personality	0.482	0.600	0.394	0.453	0.497	0.911			
7. Utilitarian value	0.541	0.458	0.515	0.643	0.644	0.445	0.920		
8. Satisfaction	0.610	0.562	0.547	0.703	0.713	0.540	0.726	0.918	
9. WOM	0.512	0.445	0.475	0.662	0.659	0.519	0.798	0.759	0.948

Furthermore, as illustrated in Table 5, all values of HTMT were below the suggested threshold of 0.90^84^, verifying discriminant validity. Thus, the measurement model demonstrated good reliability, convergent validity, and discriminant validity, indicating that our measurement scales were reliable and valid for further analysis.

**Table 5:** HTMT matrix.

Constructs	1	2	3	4	5	6	7	8	9
1. System update									
2. Memorability	0.667								
3. Non-language barriers	0.509	0.614							
4. Knowledge acquisition	0.675	0.594	0.529						
5. Knowledge application	0.767	0.711	0.613	0.847					
6. Human-like personality	0.536	0.684	0.445	0.507	0.576				
7. Utilitarian value	0.594	0.506	0.579	0.709	0.738	0.489			
8. Satisfaction	0.675	0.630	0.615	0.781	0.821	0.595	0.794		
9. WOM	0.556	0.485	0.524	0.721	0.745	0.561	0.859	0.817	

The assessment of the model fit in our study was conducted using several key fit indices, providing an overall evaluation of how well our proposed model represents the data. The fit indices include the standardized root mean square residual (SRMR), Unweighted Least Squares Discrepancy (d_ULS), Geodesic Discrepancy (d_G), Chi-square, and normed fit index (NFI). SRMR is a measure of the average discrepancy between the observed correlations and the model's predicted correlations. In our study, the SRMR values for the saturated and estimated models were 0.042 and 0.071, respectively. According to Hu and Bentler^85^, an SRMR value below 0.08 indicates a good fit, suggesting our model achieves an acceptable fit with the data. The d_ULS and d_G are discrepancy functions, with lower values indicating better model fit. Our model shows d_ULS values of 0.770 for the saturated model and 2.194 for the estimated model, and d_G values of 0.555 and 0.616, respectively. Chi-square is a traditional measure of model fit, with a lower Chi-square indicating a better fit. Our model presents Chi-square values of 1196.856 for the saturated model and 1192.325 for the estimated model. The NFI compares the fit of the target model to a null model. NFI values close to 1 indicate a better fit. In our study, both the saturated and estimated models yield an NFI of 0.857, suggesting a good fit.

### Structural model

**Figure 2:**
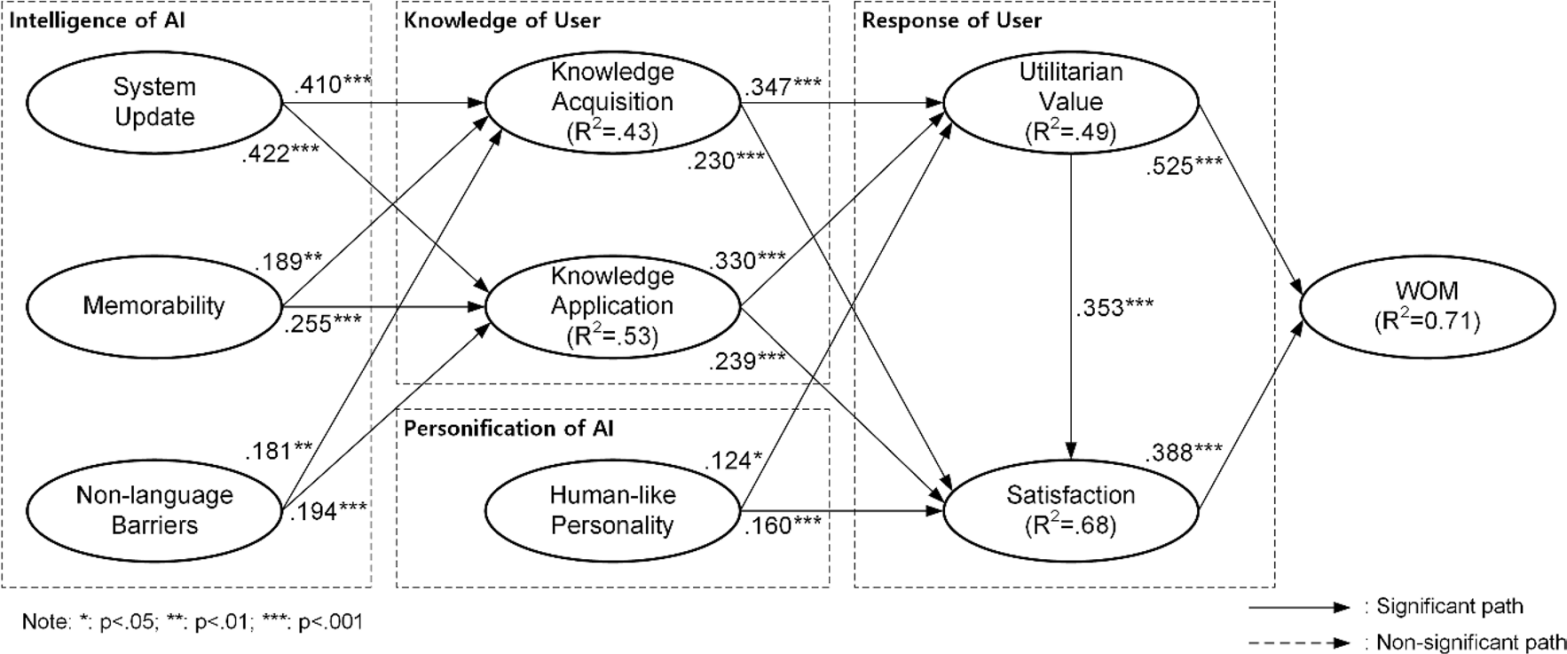
The Path Coefficients of the Research Model.

The structural model was assessed to verify the proposed hypotheses in our study. In the analysis, bootstrapping with 5000 resamples was performed to generate the t-values, p-values, and confidence intervals, which were used to determine the significance of the path coefficients^77^. Figure 2 illustrates the test results of the structural model.

The results of the structural model analysis, including the path coefficients, *t*-values, and *p*-values, are presented in Table 6. Supporting our predictions, we found significant associations between system update and both knowledge acquisition (*b* = 0.41, *t* = 6.231) and knowledge application (*b* = 0.422, *t* = 7.424), thereby confirming H1a and H1b. Similarly, memorability had a significant positive impact on both knowledge acquisition (*b* = 0.189, *t* = 2.876) and knowledge application (*b* = 0.255, *t* = 4.589), offering support for H2a and H2b. The data also indicated a significant correlation between non-language barriers and both knowledge acquisition (*b* = 0.181, *t* = 2.841) and knowledge application (*b* = 0.194, *t* = 3.312), which validates H3a and H3b. Moving on, we found that knowledge acquisition exerted a significant positive effect on both utilitarian value (*b* = 0.347, *t* = 4.466) and satisfaction (*b* = 0.23, *t* = 4.249), in line with H4a and H4b. Consistent with our hypothesis, knowledge application was significantly associated with both utilitarian value (*b* = 0.33, *t* = 4.487) and satisfaction (*b* = 0.239, *t* = 4.038), corroborating H5a and H5b. As anticipated, human-like personality positively affected both utilitarian value (*b* = 0.124, *t* = 2.429) and satisfaction (*b* = 0.16, *t* = 3.567), supporting H6a and H6b. Further, our predictions regarding utilitarian value's relationship with both satisfaction (*b* = 0.353, *t* = 5.968) and WOM (*b* = 0.525, *t* = 10.783) were strongly validated, endorsing H7a and H7b. Satisfaction was found to have a significant positive impact on WOM (*b* = 0.388, *t* = 7.432), which supports H8. In terms of control variables, gender did not show a significant influence on WOM (*b* = 0.057, *t* = 1.000), while age did (*b* = 0.060, *t* = 2.100).

**Table 6:** Results of Structural Model.

H	Cause	Effect	Coefficient	*T*-value	*P*-value	Hypothesis
H1a	System update	Knowledge acquisition	0.410	6.231	0.000	Supported
H1b	System update	Knowledge application	0.422	7.424	0.000	Supported
H2a	Memorability	Knowledge acquisition	0.189	2.876	0.004	Supported
H2b	Memorability	Knowledge Application	0.255	4.589	0.000	Supported
H3a	Non-language barriers	Knowledge Acquisition	0.181	2.841	0.005	Supported
H3b	Non-language barriers	Knowledge application	0.194	3.312	0.001	Supported
H4a	Knowledge acquisition	Utilitarian value	0.347	4.466	0.000	Supported
H4b	Knowledge acquisition	Satisfaction	0.230	4.249	0.000	Supported
H5a	Knowledge application	Utilitarian value	0.330	4.487	0.000	Supported
H5b	Knowledge application	Satisfaction	0.239	4.038	0.000	Supported
H6a	Human-like Personality	Utilitarian value	0.124	2.429	0.015	Supported
H6b	Human-like Personality	Satisfaction	0.160	3.567	0.000	Supported
H7a	Utilitarian value	Satisfaction	0.353	5.968	0.000	Supported
H7b	Utilitarian value	WOM	0.525	10.783	0.000	Supported
H8	Satisfaction	WOM	0.388	7.432	0.000	Supported
CV	Gender	WOM	0.057	1.000	0.317	Not Significant
CV	Age	WOM	0.060	2.100	0.036	Significant

*CV* stands for control variable.

*R*^*2*^ values were used to evaluate the predictive accuracy of the dependent constructs in the structural model. Collectively, the conceptual framework explained approximately 70.9 percent of the variation in WOM, indicating a substantial amount^77^.

The *Q*^*2*^ values, calculated using the PLSpredict procedure in SmartPLS 4^86^, were used to measure the predictive relevance of the model^87,88^. The Q^2^ predict values in the table represent the predictive relevance of our path model, as suggested by Hair et al.^77^. Predictive relevance measures how well the model can predict the data points for a particular endogenous latent variable. The Q^2^ predict values for all the endogenous constructs (knowledge acquisition, knowledge application, utilitarian value, satisfaction, and WOM) are greater than zero, which indicates our model has predictive relevance^77^ as shown in Table 7. These *Q*^*2*^ predict values provide evidence that our model is not only significant but also capable of accurately predicting outcomes related to these constructs.

**Table 7:** *Q*^*2*^ Value.

Construct	*Q*^*2*^ predict
Knowledge acquisition	0.415
Knowledge application	0.522
Utilitarian value	0.369
Satisfaction	0.492
WOM	0.351

Finally, the effect sizes (*f*^*2*^) were calculated to assess the substantive impact of each predictor construct on the respective endogenous construct. Table 8 provides the *f*^*2*^ effect size matrix for the constructs in our model. As per Cohen^89^, an *f*^*2*^ value of 0.02, 0.15, and 0.35 are suggestive of a small, medium, and large effect, respectively. These findings indicate that while some predictors had a more pronounced impact on certain endogenous variables, others contributed more modestly, highlighting the nuanced interplay of different factors within our model.

**Table 8:** F^2^ Matrix.

Constructs	1	2	3	4	5	6	7	8	9
1. System update				0.185	0.240				
2. Memorability				0.035	0.079				
3. Non-language Barriers				0.040	0.056				
4. Knowledge Acquisition							0.109	0.068	
5. Knowledge Application							0.094	0.070	
6. Human-like Personality							0.022	0.056	
7. Utilitarian Value								0.196	0.438
8. Satisfaction									0.242
9. WOM									

## Discussion

This section delves into a detailed analysis of the study's findings, offering insights into how each result contributes to achieving our aim: exploring the impacts of various factors, including system update, memorability, and non-language barriers, on knowledge acquisition and application, and assessing how this understanding influences user perception and experience with ChatGPT.

The current study's results affirmed the positive influence of system updates on both knowledge acquisition and application, echoing the findings of previous research^38,40,42^. This is indicative of the fact that when ChatGPT, as an AI language model, receives system updates, it enhances its capabilities to provide more accurate and extensive information, hence positively impacting the users' ability to acquire new knowledge. Moreover, system updates also seem to have a positive effect on knowledge application. As the system upgrades, it may introduce new features or improved ways of interacting with the information. For instance, new tools for organizing, filtering, or visualizing data can enhance users' ability to apply the knowledge they acquired. Therefore, as ChatGPT continually evolves, users are likely to derive more value from their interactions with it.

The significant association between memorability and both knowledge acquisition and application further strengthens previous findings that efficient recall mechanisms in AI systems can augment user experience^14^ and learning outcomes^44,45^. The seamless recall of user preferences and requirements allows for personalized interactions, consequently fostering learning and knowledge utilization.

Consistent with prior research^49^, the current research substantiated the influence of non-language barriers on knowledge acquisition and application. This indicates that as ChatGPT consistently remembers user inputs and preferences, it can provide more tailored and relevant information, enhancing the user's ability to acquire new knowledge. This capability of the system to “remember” contributes to a more personalized interaction, aligning with the user's specific needs, and can increase the efficiency of information retrieval. Furthermore, the memorability aspect also facilitates the application of knowledge. As ChatGPT remembers previous inputs, users can rely on it for continuity in their tasks, enabling them to use their acquired knowledge more effectively. For example, users can build upon previous interactions, taking the learning from past sessions and applying it to new tasks or problems.

The present study aligns with previous works^50,51^ by highlighting the significant correlation between knowledge acquisition and both utilitarian value and satisfaction. This underscores that as users acquire knowledge from their interactions with ChatGPT, they perceive an increase in practical or utilitarian value. This could be because, as users gain knowledge, they can apply it in practical contexts, leading to better decision-making, problem-solving, or task efficiency, which collectively enhance the perception of value derived from the AI system. In terms of satisfaction, the increased knowledge acquisition appears to enhance the user's contentment with ChatGPT. This could be because gaining knowledge enhances the user's self-efficacy, leading to a sense of competence and accomplishment, which in turn increases satisfaction^52,90^. This finding echoes earlier studies that have found a positive relationship between knowledge acquisition and user satisfaction in technological contexts^91^.

Our study discovered that knowledge application significantly impacts utilitarian value and satisfaction. On one hand, the utilitarian value appears to be enhanced when users can apply the knowledge acquired from ChatGPT in practical, work-related contexts. As users effectively apply the knowledge to solve problems, make informed decisions, or improve task efficiency, they perceive a higher value in using the AI system. This seems consistent with past research, which has emphasized the importance of the practical application of knowledge in improving task performance and perceived utilitarian value^92,93^. On the other hand, the users' satisfaction with ChatGPT seems to increase when they can effectively apply the knowledge gained from their interactions with the AI. This could be because the application of knowledge enhances users' sense of self-efficacy and competence, leading to an overall positive experience and greater satisfaction with the AI system^90^. This echoes the findings of prior studies that have found a positive relationship between knowledge application and user satisfaction in digital platforms^94^.

The analysis validated the significance of ChatGPT's human-like personality of utilitarian value and satisfaction. When discussing utilitarian value, it appears that interactions with an AI system that exhibits human-like personality traits enhance the perceived usefulness and practical value of the AI system for the users. This is possibly due to users finding the interaction more engaging, relatable, and user-friendly. In essence, a more "human" AI seems to offer a more valuable and meaningful interaction experience, hence increasing the perceived utilitarian value. This aligns with previous studies suggesting that human-like characteristics in AI systems enhance the perceived utility and usability of these systems^33,58^. When we turn to satisfaction, the study revealed that the human-like personality of ChatGPT also positively impacts user satisfaction. This could be attributed to the fact that interactions with a human-like AI create a sense of familiarity and comfort, thereby leading to a more satisfying user experience. This finding supports the work of Nass and Moon^95^, who suggested that users tend to respond more positively and feel more satisfied when interacting with computer agents that display human-like characteristics.

The strong correlation between utilitarian value, satisfaction, and WOM, as revealed in the current study, mirrors Lee and Kim^63^ and Mishra et al.^33^ findings. This emphasizes the critical role of perceived value in generating user satisfaction and positive WOM. When users perceive a high utilitarian value in using ChatGPT, which includes factors such as value for money, beneficial outcomes, and a sense of worthiness, their satisfaction with the AI system tends to increase. This suggests that the practical benefits that office workers gain from ChatGPT, such as productivity enhancement, task simplification, or time-saving, significantly contribute to their overall satisfaction. As for WOM, the data indicates that the higher the utilitarian value that users derive from ChatGPT, the more likely they are to spread positive WOM about it. This could be interpreted as users wanting to share and recommend useful and effective tools to their peers, colleagues, or social networks. In essence, when users perceive ChatGPT as providing substantial utilitarian value, they are inclined to talk positively about it to others, thereby promoting the AI system. This finding echoes the studies of Cheung and Thadani^8^ and Bambauer-Sachse and Mangold^96^, which concluded that the perceived utilitarian value of a product or service significantly influences positive WOM.

The study's findings corroborate the hypothesis that satisfaction with ChatGPT has a positive impact on WOM communication among office workers. This relationship between satisfaction and WOM is well-documented in the literature^67–69,97^. Studies by Anderson^69^ and Zeithaml et al.^97^ indicate that a high level of satisfaction leads to positive WOM, reaffirming the strength and relevance of this association across different fields. Essentially, when users are satisfied with the system, they are more likely to recommend it to others, thus creating a positive cycle of adoption and use.

Lastly, the results of the study revealed that while gender does not significantly influence WOM communications about ChatGPT, age does have a significant positive effect on WOM. This implies that both males and females are equally likely to share their experiences about a product or service, such as ChatGPT. On the other hand, the positive relationship between age and WOM can be understood from the perspective of life stage theory and accumulated experience. Older individuals, due to their broader life experiences and more diverse networks, may be more prone to sharing their experiences and opinions with others. Furthermore, older individuals may value the interpersonal connections facilitated by WOM more than their younger counterparts.

## Conclusion

### Implications for researchers

The findings of this study make several significant contributions to the existing body of knowledge in the field of AI systems, particularly chatbots, and their impact on the behaviors and perceptions of office workers. The first significant theoretical contribution of this study lies in its examination of the impact of system updates on knowledge acquisition and application among office workers using ChatGPT. Previous studies have shed light on how system updates can enhance user experience and satisfaction^14,36^, but this work extends the literature by focusing on knowledge-related outcomes. The findings suggest that as the system updates and becomes more sophisticated, users can acquire and apply knowledge more effectively, thus enhancing their work productivity and performance. This relationship, although logically intuitive, had not been thoroughly investigated in the context of AI chatbots before our study. As such, we contribute to the existing body of knowledge by revealing how continuous system improvements can enhance users' learning and application abilities, thereby driving value creation in an office environment. This helps fill a critical gap in the literature on AI chatbots and their role in knowledge management, thus providing scholars with a new perspective to further explore.

Secondly, the study highlighted the role of memorability in knowledge acquisition and application, another underexplored area in prior research. While existing literature has explored aspects of memorability in user interfaces and experiences^98,99^, few have delved into its role in AI chatbot contexts. The results demonstrate that ChatGPT's ability to remember user preferences and requirements significantly enhances knowledge acquisition and application, pointing to the importance of developing AI systems with high memorability. For researchers in the field, this study sets the foundation for a more detailed exploration of the significance of memorability as a crucial component of the user experience with AI systems. Scholars could further investigate how the perceived memorability of AI systems influences other aspects of the user's experience, like trust in the system or overall satisfaction. Such research could yield important insights into designing AI chatbots for effective knowledge management in the workplace and beyond. Furthermore, the findings could lead to the exploration of the long-term impacts of memorability on the users' continuous use of AI systems, or how it could shape the dynamics of collaborative work where such systems are utilized.

Thirdly, this study extends the understanding of non-language barriers in the context of AI chatbots. Prior studies have indicated that language could be a barrier to technology use^100,101^, but the focus has not been on how overcoming this barrier, particularly in the context of office workers, could affect knowledge acquisition and application. Our study addresses this gap by suggesting that the ability of AI chatbots to effectively communicate across different languages can positively influence the process of knowledge acquisition and application. This offers new insights into the importance of language versatility in AI chatbots, particularly in diverse and multilingual work environments. For scholars, our findings suggest the need to further explore how non-language barriers in AI systems could impact other knowledge-related processes and outcomes in the workplace. This could extend to understanding how such barriers in AI communication could influence collaborative work processes, decision-making, or innovation, thereby opening up new frontiers in AI research within the workplace context.

The fourth contribution lies in the exploration of robust empirical validation for the influence of knowledge acquisition and application through the use of AI chatbots like ChatGPT in the workplace. Previous research has suggested that AI can contribute significantly to knowledge management within an organization^102–104^, but our study goes a step further by dissecting this effect into the distinct domains of knowledge acquisition and application. Existing literature largely focuses on the general benefits of knowledge acquisition^25,105,106^ and knowledge application^107–109^, but the direct link to utilitarian value and satisfaction in the context of AI chatbots remains underexplored. This research empirically demonstrates the impact of knowledge acquisition and application on utilitarian value and satisfaction, an aspect not adequately covered in prior studies. From a theoretical perspective, these findings encourage scholars to consider the distinct but interconnected roles of knowledge acquisition and application in the context of AI use in the workplace. Our findings suggest that the way this knowledge is subsequently applied could be just as important, if not more so, for driving value and satisfaction. Future research could further delve into these aspects, possibly looking into how different types of knowledge or different application methods could have varying impacts on user outcomes. This study also calls attention to the need for further exploration of the intricate dynamics between various chatbot attributes and the specific domains of knowledge acquisition and application. For example, it would be worthwhile to investigate whether certain system update features are particularly beneficial for knowledge acquisition, while others have a more significant impact on knowledge application. As the capabilities of AI systems continue to evolve, scholars need to keep pace with these advancements and continuously reassess their impacts on knowledge management in the workplace.

Lastly, this study advances the understanding of how human-like personality in AI systems influences utilitarian value and satisfaction. Prior research has acknowledged the importance of perceived personality in AI systems^95,110–113^, but the current study delves deeper by connecting these characteristics to tangible outcomes like utilitarian value and satisfaction within an AI chatbot context. This specific finding provides a novel perspective, enhancing the theoretical understanding of the relevance of human-like qualities in AI systems. By identifying the human-like personality of a chatbot as a significant determinant of utilitarian value and satisfaction, we invite scholars to explore further how users perceive and interact with AI systems that display human-like traits. For instance, subsequent studies could investigate how specific human-like characteristics (e.g., empathy, humor, emotional intelligence) individually contribute to perceived value and satisfaction. Our findings also suggest the necessity for a more nuanced understanding of how different user groups may react to human-like AI personalities. Do all users equally appreciate these traits, or do some find them discomforting or irrelevant to their utilitarian objectives? Continued investigation in this domain would refine the current knowledge base regarding user preferences and expectations with AI personality design.

### Practical implications

The current study's findings present valuable practical implications for various stakeholders, including managers, developers, marketers, and users of AI-based chatbots such as ChatGPT.

The first vital practical implication of the present study's findings concerns business managers, particularly those responsible for investing in and managing AI-based tools like ChatGPT within an organizational context. Central to this role is the understanding of the importance of system updates in enhancing the knowledge acquisition and application capabilities of office workers, a relationship substantiated by the present study. In today's dynamic technological landscape, it's essential to ensure that AI tools are regularly updated to integrate the latest developments and capabilities^114^. These updates can significantly enhance the utility of ChatGPT as an informational resource, improving its capacity to support knowledge acquisition and application among office workers. As such, managers should prioritize investments in these regular system updates, viewing them not as optional enhancements, but as fundamental necessities to ensure the tool remains at the forefront of technology.

The second practical implication of our findings targets developers in the AI industry, focusing specifically on how they might enhance the utility and usability of chatbots like ChatGPT. Our findings highlight the importance of two key areas in chatbot development—enhancing memorability and mitigating non-language barriers. Memorability not only enriches the user's interaction with the chatbot but also allows for a personalization that mimics human-like memory retention and recall^114^. As our findings have illustrated, features that enhance the memorability of a chatbot significantly impact users' knowledge acquisition and application. For instance, a chatbot that remembers a user's previous queries can provide more contextually relevant and accurate responses. Such an approach will be particularly effective in office settings where users interact with the chatbot on a range of issues over time. On the other hand, addressing non-language barriers is an essential aspect of the development of chatbots, especially for use in multicultural office environments. Non-language barriers may include elements such as culture, emotion, context, and non-verbal cues^14^. Our study's results suggest that addressing these barriers positively impacts knowledge acquisition and application. Developers can thus focus on enhancing features that enable a more seamless transition across various cultural contexts, incorporating emotional understanding, and context awareness. Such features could significantly improve the user experience, especially in diverse and multicultural work environments. In essence, our results validate the importance of these features in the development process. Therefore, developers should prioritize enhancing these aspects of chatbot design to ensure a richer, more immersive, and inclusive user experience.

The third practical implication pertains to the realm of marketing. Marketers, with their crucial role in shaping the public perception and appeal of products like ChatGPT, can leverage the findings from this study to refine their promotional strategies. Our study revealed that the human-like personality of the AI chatbot and its ongoing system updates significantly impact its perceived utilitarian value and user satisfaction. This insight provides marketers with a focused approach to positioning their chatbot. By accentuating the chatbot's human-like personality in promotional materials, marketers can appeal to the psychological aspects of consumer decision-making^112^. A human-like personality in AI systems can create a sense of familiarity and affinity, leading to an increased likelihood of adoption and continuous usage. In addition, highlighting the chatbot's commitment to regular system updates underscores the product's promise of innovation and continuous improvement. Office workers, who often value practicality and efficiency, are likely to appreciate a tool that remains at the forefront of technological advancements and is always evolving to cater to their needs better^115^. Thus, emphasizing this feature can appeal to their preference for utilitarian gains and may enhance both user acquisition and engagement. In sum, marketers can strategically use the insights from our study to highlight the chatbot's human-like personality and commitment to system updates, thereby enhancing its market appeal and user experience.

The final implication pertains to end-users, particularly office workers who form the target user group of AI chatbots such as ChatGPT. Our findings underscore how these users can maximize the benefits derived from the use of such technologies. Given the significant influence of system updates on knowledge acquisition and application demonstrated by our results, users can optimize their learning and productivity by leveraging a chatbot that undergoes frequent system updates. By choosing and sticking with a chatbot that continuously improves and stays at the cutting edge of technology, office workers can ensure they are always accessing the most accurate and comprehensive information available, thereby facilitating their knowledge acquisition. Further, our study highlights the importance of memorability and non-language barrier features in AI chatbots. Hence, users can gain more value by utilizing chatbots that remember their preferences, which can enhance the user experience by making the interaction more personalized and efficient^116^. Similarly, chatbots that can accommodate multilingual users can be particularly valuable in multicultural business settings, ensuring no user is left behind due to language barriers^19,117^. By harnessing an AI chatbot with these key features, office workers can significantly enhance their knowledge acquisition and application, which in turn can boost their productivity and job satisfaction. This can, therefore, be a key strategy for office workers aiming to harness the power of AI to augment their work processes.

### Limitation and further research

While the current study provides several insightful contributions, it is not devoid of limitations that offer avenues for future research. One of the distinctive limitations of this study is the use of self-reported measures for evaluating knowledge acquisition and application, which may be subject to social desirability bias. Future studies could incorporate more objective measures such as performance-based tasks or observation methods to assess the actual impact of ChatGPT on knowledge processes. The research also focused predominantly on office workers as the user group. Though this emphasis has its merits, it may limit the generalizability of the findings. Future research could explore the influence of ChatGPT and similar AI chatbots on different demographic groups such as students, seniors, or specific professional categories (e.g., medical, legal) to enrich our understanding of AI chatbot utilization in diverse contexts.

Additionally, this study only explored a limited set of potentially influential factors. Future work could delve into other factors such as trust, perceived ease of use, aesthetics, or user traits that might also significantly influence user interaction with AI chatbots. Specifically, future studies should consider a wider range of user traits, such as user innovativeness, prior knowledge, and self-efficacy. These factors are critical in understanding how individuals engage with and derive satisfaction from technology-oriented services like ChatGPT. Investigating these traits will provide a more comprehensive understanding of user behavior and interaction with AI chatbots. Given the nature of ChatGPT as an AI-assisted content creation tool, future research must address the ethical implications, particularly in terms of copyright. This area requires careful exploration to understand how users perceive and navigate the ethical considerations involved in using AI for content generation. Furthermore, the context in which ChatGPT is used is another vital area for future research. Factors such as the difficulty, creativity, and analytical or intuitive nature of the task at hand may influence how users perceive and interact with ChatGPT. Future studies should explore how these contextual variables affect user experience and outcomes.

Moreover, one limitation of our study is the narrow focus on personification without extensively exploring its interplay with the independent variables, which could provide a more holistic understanding of AI interaction. Future research could delve deeper into how system updates, memorability, and non-language barriers contribute to both AI personification and personalization, thereby enhancing our comprehension of AI-user dynamics in the workplace.

Finally, investigating ChatGPT's potential in reducing user stress is an intriguing avenue for future research. Even if ChatGPT does not directly solve a problem, the mere presence of an AI knowledge mentor could potentially alleviate stress during problem-solving processes. Future studies could examine the psychological impacts of interacting with AI chatbots like ChatGPT.

## Data Availability

The datasets used and/or analyzed during the current study available from the corresponding author on reasonable request.
